# A comparative study of genotyping and antimicrobial resistance between carbapenem-resistant *Klebsiella pneumoniae* and *Acinetobacter baumannii* isolates at a tertiary pediatric hospital in China

**DOI:** 10.3389/fcimb.2024.1298202

**Published:** 2024-03-08

**Authors:** Xiaoli Jian, Yunyun Li, Haiping Wang, Cuilian Li, Feng Li, Jue Li, Jing Dong, Tingyi Du, Li Jiang

**Affiliations:** Kunming Children’s Hospital (Kunming Medical University Affiliated), Kunming, Yunnan, China

**Keywords:** carbapenem-resistant *Klebsiella pneumoniae*, carbapenem-resistant *Acinetobacter baumannii*, REP-PCR, carbapenemase, pediatric patient

## Abstract

**Background:**

Carbapenem-resistant *Klebsiella pneumoniae* (CRKP) clinical isolations have rapidly increased in pediatric patients. To investigate a possible health care-associated infections of CRKP in a tertiary pediatric hospital, the circulating clones and carbapenem-resistant pattern between CRKP and carbapenem-resistant *Acinetobacter baumannii* (CRAB) isolates were compared to classify their epidemiological characteristics. The results will help to identify the epidemic pattern of the CRKP transmission in the hospital.

**Methods:**

Ninety-six CRKP and forty-eight CRAB isolates were collected in Kunming Children’s Hospital from 2019 through 2022. These isolates were genotyped using repetitive extragenic palindromic-PCR (REP-PCR). Carbapenemase phenotypic and genetic characterization were investigated using a disk diffusion test and singleplex PCR, respectively. In addition, these characteristics of the two pathogens were compared.

**Results:**

The rates of CRKP and CRAB ranged from 15.8% to 37.0% at the hospital. Forty-nine and sixteen REP genotypes were identified among the 96 and 48 CRKP and CRAB isolates tested, respectively. The CRKP isolates showed more genetic diversity than the CRAB isolates. Of the 96 CRKP isolates, 69 (72%) produced Class B carbapenemases. However, all 48 CRAB isolates produced Class D carbapenemase or extended-spectrum β-lactamases (ESBL) combined with the downregulation of membrane pore proteins. Furthermore, the carbapenemase genes *bla*_KPC_, *bla*_IMP_, and *bla*_NDM_ were detected in CRKP isolates. However, CRAB isolates were all positive for the *bla*_VIM_, *bla*_OXA-23_, and *bla*_OXA-51_ genes.

**Conclusions:**

These CRKP isolates exhibited different biological and genetic characteristics with dynamic changes, suggesting widespread communities. Continuous epidemiological surveillance and multicenter research should be carried out to strengthen the prevention and control of infections.

## Introduction

Infections caused by multidrug-resistant organisms (MDROs) have been identified as a top global public health concern, with particular attention to *Klebsiella pneumonia* (World Health Organization (WHO), 2014; Ding et al., 2023). Through genetic mutation and the acquisition of mobile genetic elements (Beatson and Walker, 2014), the pathogen has developed resistance mechanisms against oxazolidinones, lipopeptides, macrolides, and antibiotics that are the last line of defense, including carbapenems (Zaman et al., 2017). According to the China Antimicrobial Resistance Surveillance Trial (CARST) program, the frequency of carbapenem-resistant *K. pneumonia* (CRKP) clinical isolations has rapidly increased from 2.9% in 2005 to 24.2% in 2022 (CHINET(ChinaAntimicrobial Surveillance Network), 2023). Therefore, the high detection rate of carbapenem resistance brings a dilemma of no medication available to clinicians.

Identification of risk factors of CRKP infection is critical for informing the prevention and control of CRKP spreading. Intensive Care Unit (ICU) admission, respiratory failure, carbapenems use, central venous catheter use, and colonization with CRKP, were independent predictors for acquisition (Gallagher et al., 2014; Gómez Rueda and Zuleta Tobón, 2014; Huang et al., 2023). However, these risk factors were varied in geography and population. Our previous study demonstrated that re-hospitalization, carbapenems use, and invasive procedure, were independent risk factors for CRKP infection among children who visited at Kunming Children’s Hospital (Dai et al., 2022). The finding indicated possible health care-associated infections in the hospital.

To further confirm this finding, carbapenem-resistant *Acinetobacter baumannii* (CRAB), the most common nosocomial pathogen (Kurihara et al., 2020), was employed in the study. The circulating clones and carbapenem-resistant pattern between CRKP and CRAB isolates were compared. These results will help to identify the epidemic pattern of the CRKP transmission in the hospital.

## Methods

### Study setting

The study was conducted in Kunming Children’s Hospital, a tertiary hospital with more than 1200 beds and annual capacity of more than 2,000,000 outpatients, integrated with health care, teaching, scientific research for children living in Kunming City, Yunnan Province, China. Collection of the isolates and their examination was approved by the institutional ethical review board at the Kunming Children’s Hospital (No. 2022-03-175-K01).

### Bacterial identification and antimicrobial susceptibility testing

*Klebsiella pneumoniae* and *Acinetobacter baumannii* were aseptically isolated from blood, ascites, drainage fluid, cerebrospinal fluid, lower respiratory tract secretions and other patient samples, and then identified to species level through an automatic microbial identification platform (BacT/AlerT 3D, BioMérieux, Marcy l’Etoile, France) within the hospital clinical laboratory according to the manufacturers’ instructions.

Antimicrobial susceptibility testing of the isolates to common clinically used antibiotics was performed via the broth micro-dilution method using an automated VITEK 2 Compact system with Advanced Expert System (BioMérieux, Marcy l’Etoile, France) approach according to the Clinical and Laboratory Standards Institute (CLSI) unified protocol (Weinstein and Lewis, 2020). The VITEK 2-Compact uses the companion GN334 and GN335 antimicrobial susceptibility cards for *Klebsiella pneumoniae* and *Acinetobacter baumannii*, respectively. *Escherichia coli* ATCC25922 were used as quality controls.

### Study strains

A total of nonduplicated 191 CRKP and 99 CRAB isolates were recovered from routine microbiological laboratory procedures at Kunming Children’s Hospital from 2019 through 2022 (**Table 1**). These clinical isolates had been frozen at -20°C using strain store medium (Hopebio, Qingdao, China). Subsequent re-culturing was performed by sub-culturing a loop-full of the frozen stock on to blood agar (Autobio, Zhenzhou, China), and checked for purity by examining colony color and morphology. At last, 96 CRKP and 48 CRAB isolates were successfully rescued and used for the comparative studies.

**Table 1:** Carbapenem-resistant profiles of clinical isolated *K. pneumonia* and *A. baumannii* at Kunming Children’s Hospital from 2019 through 2022.

Year	Number of *K. pneumonia* isolates	Number of CRKP isolates	Carbapenem-resistant rate %	Number of CRKP isolates in the study	Number of *A. baumannii* Isolates	Number of CRAB isolates	Carbapenem-resistant rate %	Number of CRAB isolates in the study
2019	217	61	28.1	3	71	24	33.8	2
2020	184	29	15.8	20	96	34	36.6	13
2021	146	34	23.4	25	54	20	37.0	12
2022	266	67	24.5	48	62	21	33.9	21
total	813	191	23.5	96	283	99	35.0	48

### DNA template preparation

From a fresh overnight culture plate on blood agar, 4-5 colonies were removed using a sterile toothpick and inoculated into a 0.2 mL PCR tube containing nuclease free water. The tubes were vortexed for approximately 30 s to suspend the cells. After suspension, the tubes were boiled at 95°C for 15 min in a dry thermostat K30 (Allsheng, Hangzhou, China) to lyse the cells. After boiling, the samples were frozen in −80°C freezers during PCR reagent preparation to enhance lysis of any remaining intact cells.

### Repetitive Element Palindromic (REP)-PCR genotyping

Due to REP-PCR is economical and efficient method among nosocomial infection identification (Viau et al., 2017). The method was performed using the primers and cycling conditions (**Supplementary Table 1**). The oligonucleotide primers were prepared by the Kunming University of Science and Technology. Amplification reactions were performed using Promega’s GoTaq R Green Master Mix (TAKARA, Madison, Japan). Each reaction contained 12.5µL of GoTaq R Green, 2µL of template, 1µL of 1µM primer mixture of both forward and reverse primers, and 9.5µL of nuclease-free water. A 2 kb Plus DNA ladder was used every 11 lanes to ensure comparability between lanes (**Supplementary Figure 1**). Phylogenetic trees were constructed using Bionumerics version 8.0. Lanes and bands were manually detected and compared to a 2 kb plus ladder lane. The neighbor joining method was used to visually display REP-clusters (**Supplementary Figure 2**).

### Detection of carbapenemase phenotypic characterization

Class A and B carbapenemases expressed by the selected CRKP and CRAB isolates were subjected to phenotypic characterization using a disk diffusion test kit (DL Biotech, Zhuhai, China) as described by Chinese expert consensus (Hua et al., 2022). In briefly, three combined-disc tests using discs of imipenem (IPM) alone and with ethylene diamine tetraacetic acid (EDTA), aminoethyl di phenylboronic acid (APB), or EDTA plus APB was tested for each isolate. Production of class A carbapenemase was considered when the growth-inhibitory zone diameter around the IPM disc with APB and the IPM disc with both APB and EDTA was increased ≥5 mm compared with the growth-inhibitory zone diameter around the disc containing IPM alone. Production of class B was considered when the growth-inhibitory zone diameter around the IPM disc with EDTA and the IPM disc with both APB and EDTA was increased ≥5 mm compared with the growth-inhibitory zone diameter around the disc containing IPM alone. Production of both class A and B enzymes was considered when the growth-inhibitory zone diameter around the IPM disc with both PBA and EDTA was increased ≥5 mm compared with the growth-inhibitory zone diameter around the disc containing IPM alone while the growth-inhibitory zone diameters around the IPM disc with PBA and the IPM disc with EDTA were increased ≥5 mm compared with the growth-inhibitory zone diameter around the disc containing IPM alone. Production of class D carbapenemase or extended-spectrum β-lactamases (ESBL) combined with downregulation of membrane pore proteins were considered when the all the three combined-disc tests was positive and the growth-inhibitory zone diameters were increase <5 mm compared with the growth-inhibitory zone diameter around the disc containing IPM alone. Finally, when none of the three combined-disc and the IPM alone disc tests was positive, the isolate was considered negative for carbapenemase production (**Supplementary Figure 3**).

### Detection of carbapenemase genes

All CRKP and CRAB clinical isolates were screened for 11 different genes encoding for class A, B, and D carbapenemases. One class A carbapenemase genes (*bla*_KPC_), six class B carbapenemase genes (*bla*_IMP_, *bla*_VIM_, *bla*_NDM_, *bla*_GIM_, *bla*_SPM_, and *bla*_SIM_), and four Class D genes (*bla*_OXA-23_, *bla*_OXA-24_, *bla*_OXA-51_, *bla*_OXA-58_) were screened using the primers and cycling conditions (**Supplementary Table 2**).

### Statistics

All statistical analyses were performed on R program (version 4.1.1). To determine whether frequency counts of genetic and biological characteristics are distributed the same across different populations of CRKP and CRAB, fisher’s exact test of homogeneity was used. All calculations used a 0.05 level of significance with a two-sided test.

## Results

### CRKP and CRAB clinical isolates

From 2019 through 2022, the annual carbapenem-resistant rate ranged from 15.8% to 37.0% among *K. pneumonia* and *A. baumannii* isolates. The carbapenem-resistant rate presented higher proportions among *A. baumannii* isolates than among *K. pneumonia* isolates (**Table 1**). A total of 96 CRKP and 46 CRAB stored isolates from the above clinical routine work were collected for subsequent analysis. The CRKP isolates were mostly from sputum and blood. However, CRAB isolates were mainly contributed from sputum and wound secretion (**Table 2**). These carbapenemase-producing strains possessed a wide range of antimicrobial agents, including aminoglycosides, fluoroquinolones, and trimethoprim-sulfamethoxazole (**Table 3**).

**Table 2:** Specimen sources of selected CRKP and CRAB isolates.

Specimen sources	CRKP n=96		CRAB n=48	
	number	proportion %	number	proportion %
Sputum	70	72.9	35	72.9
Blood	12	12.5	3	6.3
Urine	9	9.4	0	0.0
Cerebrospinal fluid	2	2.1	0	0.0
Wound secretion	1	1.0	6	12.5
Lavage fluid	1	1.0	2	4.2
Tip catheter	1	1.0	0	0.0
Feces	0	0.0	2	4.2

CRKP, carbapenem-resistant Klebsiella pneumoniae; CRAB, carbapenem-resistant Acinetobacter baumannii.

**Table 3:** Antibiotic sensitivity information for selected CRKP and CRAB.

Antibiotic	CRKP (GN334 card)						CRAB (GN335 card)					
	Sensitive		Intermediate		Resistant		Sensitive		Intermediate		Resistant	
	No.	%	No.	%	No.	%	No.	%	No.	%	No.	%
Amoxicillin/Clavulanic Acid	0	0.0	0	0.0	95	100.0	1	2.9	0	0.0	34	97.1
Trimethoprim/Sulfamethoxazole	47	49.0	0	0.0	49	51.0	9	19.15	1	2.1	37	78.7
Imipenem	0	0.0	0	0.0	96	100.0	0	0.0	0	0.0	48	100.0
Ceftazidime	0	0.0	0	0.0	96	100.0	1	2.1	0	0.0	46	97.9
Cefepime	2	2.1	0	0.0	94	98.0	0	0.0	4	8.5	43	91.5
Ceftriaxone	0	0.0	1	1.1	94	99.0	0	0.0	0	0.0	36	100.0
Aztreonam	4	16.7	0	0.0	20	83.3	0	0.0	0	0.0	35	100.0
Piperacillin/Tazobactam	0	0.0	0	0.0	96	100.0	0	0.0	0	0.0	32	100.0
Cefoperazone/Sulbactam	0	0.0	0	0.0	73	100.0	1	3.1	1	3.1	30	93.7
Levofloxacin	26	27.1	33	34.4	37	38.5	1	2.1	5	10.6	41	87.2
Tigecycline	64	97.0	0	0.0	2	3.0	22	68.7	10	31.3	0	0.0
Cefuroxime	0	0.0	0	0.0	72	100.0	–	–	–	–	–	–
Amikacin	67	69.8	0	0.0	29	30.2	–	–	–	–	–	–
Cefoxitin	0	0.0	0	0.0	72	100.0	–	–	–	–	–	–
Ertapenem	0	0.0	0	0.0	73	100.0	–	–	–	–	–	–
Cefuroxime Axetil	0	0.0	0	0.0	72	100.0	–	–	–	–	–	–

CRKP, carbapenem-resistant Klebsiella pneumoniae; CRAB, carbapenem-resistant Acinetobacter baumannii. "–" means no operation.

### REP cluster analysis

**Figure 1:**
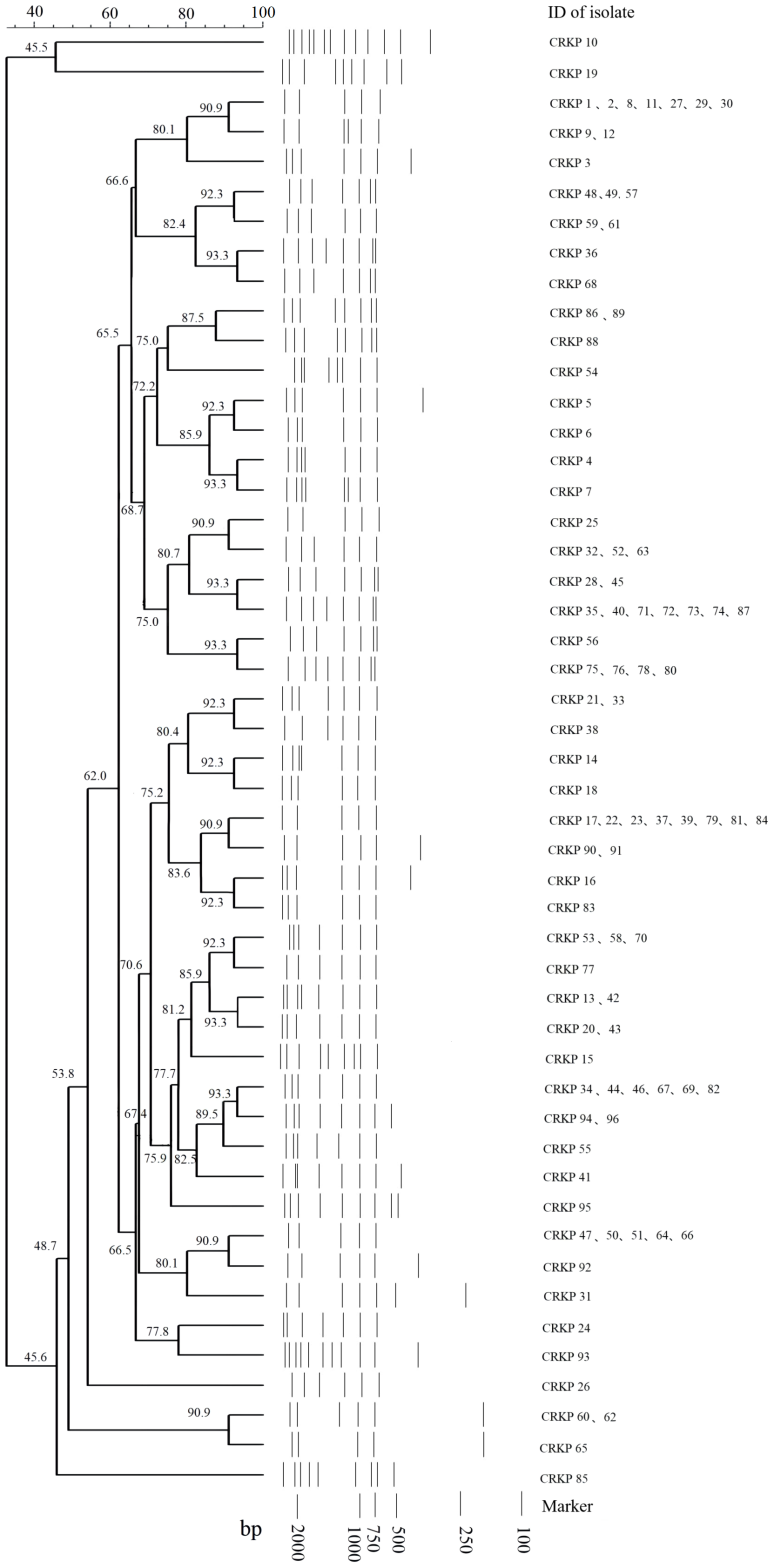
Sample DNA fingerprint generated from Repetitive Element Palindromic-PCR (REP-PCR) of carbapenem-resistant *Klebsiella pneumoniae* (CRKP) isolates collected at Kunming Children’s Hospital by date of isolation, and phylogenetic tree of sample gel using neighbor joining method.

**Figure 2:**
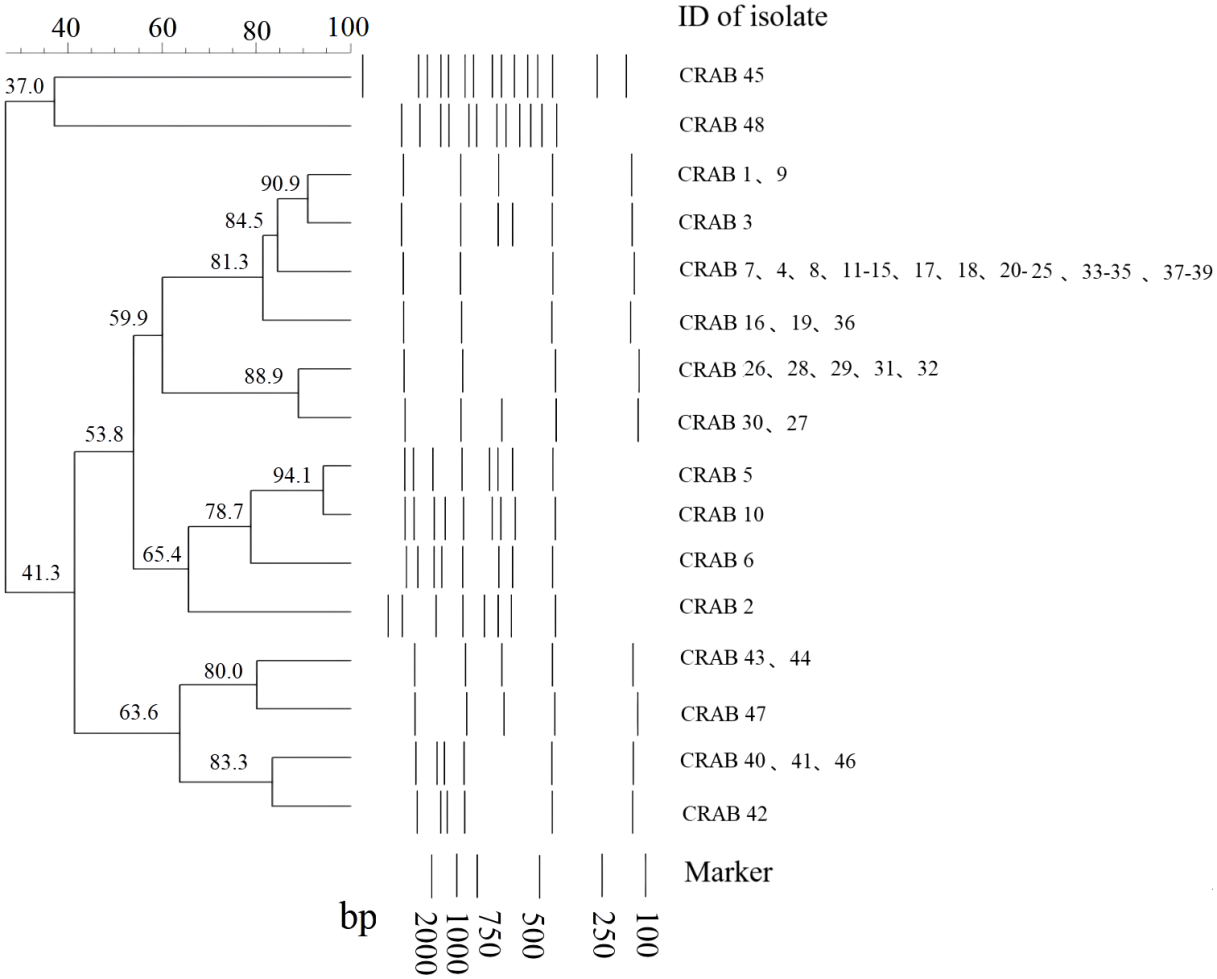
Sample DNA fingerprint generated from Repetitive Element Palindromic-PCR (REP-PCR) of carbapenem-resistant *Acinetobacter baumannii* (CRAB) isolates collected at Kunming Children’s Hospital by date of isolation, and phylogenetic tree of sample gel using neighbor joining method.

There were 49 and 16 REP genotypes identified among the 96 and 48 CRKP and CRAB isolates tested, respectively (**Figures 1**, **2**). Five genotypes were observed at least five to eight times in the CRKP collection (**Figure 1**). These cluster CRKP clones were identified sporadically by year and sample source (**Supplementary Figure 2**). However, one genotype was observed more than 10 times and accounted for 45.8% (22/48) of the CRAB collection (**Figure 2**). These same CRAB cloned strains were isolated from various clinical samples, including sputum, wound sections, and blood, throughout the surveillance years (**Supplementary Figure 2**).

### Phenotypic characteristics of carbapenemases

The phenotype-based detection results of the disk diffusion assay showed that 27, 68, and 1 CRKP isolates produced Class A, B, and A+B carbapenemases, respectively. However, all 48 CRAB isolates produced Class D carbapenemase or extended-spectrum β-lactamases (ESBL) combined with the downregulation of membrane pore proteins. Statistically, there was a significant difference in the distribution of carbapenemase patterns between the CRKP and CRAB strains (p < 0.01) (**Table 4**).

**Table 4:** Distribution of carbapenemase patterns among the selected CRKP and CRAB isolates.

Carbapenemase patterns	CRKP		CRAB		Fisher’s exact test
	n	%	n	%	*P*
A	27	28.1	0	0.0	< 0.01
B	68	70.8	0	0.0	
A+B	1	1.1	0	0.0	
D or ESBL combining	0	0.0	48	100.0	

CRKP, carbapenem-resistant Klebsiella pneumoniae; CRAB, carbapenem-resistant Acinetobacter baumannii.

### Genotypic characteristics of carbapenemases

After gene extraction, 96 CRKP and 48 CRAB isolates were examined genotypically for 11 carbapenemase genes by singleplex PCR methods. Overall, only 1 of the CRKP isolates was positive for two carbapenemase genes (*bla*_KPC_+*bla*_NDM_), while the remaining 95 isolates produced only one carbapenemase gene. The results included 27 isolates producing *bla*_KPC_, 29 isolates producing *bla*_IMP_, and 39 isolates producing *bla*_NDM_. However, all 46 CRAB isolates were positive for three carbapenemase genes (*bla*_VIM_, *bla*_OXA-23_, *bla*_OXA-51_). A statistical study verified that there was a highly significant difference in the prevalence of carbapenemase genes among the tested isolates by (p < 0.01) (**Table 5**).

**Table 5:** Genotype detection among selected CRKP and CRAB isolates.

Carbapenemase genes		CRKP		CRAB		*P*
		n	%	n	%	
A	bla_KPC_	28	29.2	0	0.0	< 0.01
B	bla_IMP_	29	30.2	0	0.0	
	bla_VIM_	0	0.0	48	100.0	
	bla_NDM_	40	41.6	0	0.0	
D	bla_OXA-23_	0	0.0	48	100.0	
	bla_OXA-51_	0	0.0	48	100.0	

CRKP, carbapenem-resistant Klebsiella pneumoniae; CRAB, carbapenem-resistant Acinetobacter baumannii.

## Discussion

This study was conducted to investigate the genetic and biological characteristics of CRKP and CRAB isolates in a tertiary pediatric hospital. Overall, the rates of CRKP and CRAB ranged from 15.8% to 37.0%. CRKP isolates showed more genetic diversity than CRAB isolates. Class A and B carbapenemases were detected among CRKP isolates, while Class D carbapenemase or ESBL combined with downregulation of membrane pore proteins was observed among CRAB isolates. Carbapenemase genes *bla*_KPC_, *bla*_IMP_, or *bla*_NDM_ were observed in CRKP isolates. However, all CRAB isolates harbored the triple resistance genes *bla*_VIM_, *bla*_OXA-23_, and *bla*_OXA-51_.

According to the Chinese Infectious Disease Surveillance of Pediatrics (ISPED) from 13 sentinel pediatric hospitals, the rates of CRKP and CRAB were 14.8% and 30.7% among children in 2021, respectively (Pan et al., 2022). The resistance rate in our study displayed a similar but lower than 24.2% and 71.9% in adults from CARST in China (CHINET(ChinaAntimicrobial Surveillance Network), 2023). The higher resistance rate in adults might be caused by selective pressure due to the lengthy usage of antibiotics in elderly patients who are vulnerable to chronic diseases.

Currently, carbapenem resistance in *A. baumannii* infections isolated from community-dwelling patients has remained lower (van Duin and Paterson, 2020). PCR fingerprinting of 29 imipenem-resistant *A. baumannii* isolates collected at a large hospital in Spain showed that they belonged to a single circulating clone (Bou et al., 2000). A similar study conducted in China found that 24 multidrug-resistant isolates collected from various sources in Sichuan Province had identical REP-PCR patterns (Luo et al., 2011). In this study, nearly half of the 48 CRAB isolates from the same genetic clade were shown to belong to the same clone and spread longitudinally, suggesting that clonal spread of CRAB might have occurred in the hospital. In contrast, CRKP has been detected in many environments, such as clinical settings, hospital wastewater, drinking water, and food-producing animals (Runcharoen et al., 2017), and has become prevalent cause of community-acquired infections (van Duin and Paterson, 2020). Our results revealed the genetic diversity of CRKP isolates without obvious aggregation in terms of sex, age, ward, and time, suggesting that they might originate from multiple sources.

For carbapenemase detection, all CRAB isolates in the study produced Class D carbapenemase or extended-spectrum β-lactamases (ESBL) combined with the downregulation of membrane pore proteins and were positive for the *bla*_VIM_, *bla*_OXA-23_, and *bla*_OXA-51_ genes. The triple-harbored resistance genes in CRAB have been reported from children’s isolates in eastern China (Zhu et al., 2022). These coharbored carbapenemase gene combinations were common among CRAB isolates and varied in geography (Huang et al., 2019; Sawant et al., 2022). Such high consistency of gene combinations in the study further confirmed our hypothesis on health care-associated infection for CRAB. However, *bla*_NDM_ was the most common carbapenemase in CRKP, followed by *bla*_IMP_ and *bla*_KPC_. Consistent with a previous report, the most prevalent carbapenemase gene at CRKP was *bla*_KPC_ among isolates from adult patients, and *bla*_NDM_ among isolates from children in China (Han et al., 2020). The prevalence and clinical importance of *bla*_NDM_ gene, which may be associated with environmental selective stress, in pediatric patients. In contrast, the *bla*_KPC_ gene, which was prevalent in Chinese adults, mainly led to CRKP infections in pediatric patients with a surgical history in general and external hospitals (Jiang et al., 2023). Thus, multicenter center study is necessary to identify the infection sources.

Our study had several limitations. First, not all isolates have been successfully rescued for molecular typing, which may have introduced selection bias to display REP-clusters. Second, only 11 carbapenemases genes were screened, some emergent resistant gene may have ignored in the investigation. However, the characteristics of both CRKP and CRAB isolates were compared in the study, which indicated the different epidemic patterns.

In conclusion, these CRKP isolates exhibited different biological and genetic characteristics with dynamic changes, suggesting widespread communities. However, most CRAB isolates shared the same biological and genetic characteristics, suggesting that intra-hospital transmission may have occurred. Therefore, whole genome sequencing (WGS) should be carried out to further confirm nosocomial transmission. Continuous epidemiological surveillance should be enhanced to monitor the dynamic changes in epidemic strains. In addition, multicenter research will be useful to understand pathogen dissemination in this region.

## Data availability statement

The original contributions presented in the study are included in the article/**Supplementary Material**. Further inquiries can be directed to the corresponding author.

## Author contributions

XJ: Data curation, Formal analysis, Investigation, Methodology, Writing – original draft. YL: Data curation, Formal analysis, Investigation, Methodology, Writing – original draft. HW: Data curation, Formal analysis, Investigation, Methodology, Writing – original draft. CL: Funding acquisition, Writing – review & editing, Methodology. FL: Methodology, Writing – review & editing. JL: Methodology, Writing – review & editing. JD: Methodology, Writing – review & editing. TD: Supervision, Writing – review & editing. LJ: Funding acquisition, Project administration, Resources, Supervision, Writing – review & editing.
