# Supplementary material for: A comparative study of genotyping and antimicrobial resistance between carbapenem-resistant Klebsiella pneumoniae and Acinetobacter baumannii isolates at a tertiary pediatric hospital in China
Source: Front Cell Infect Microbiol. 2024 Mar 8;14:1298202. doi: 10.3389/fcimb.2024.1298202 (PMC10960357; doi:10.3389/fcimb.2024.1298202)
Supplement: Supplementary file 1 [file DataSheet_1.pdf]

Supplemental Table 1 Primers and cyclic conditions used in Rep-PCR among CRKP and CRAB clinical isolates

Primer	Primer sequence	Cycling condition	Reference
REP typing	F: IIIGCGCCGICATCAGGC<br>R: ACGTCTTATCAGGCCTAC	Initial denaturation at 95℃ for 3 min, then 30 cycles of 90℃ for 30s, 40℃ for 1 min and 65℃ for 8 min. A final extension time of 16 min at 65℃.	(Vila et al., 1996)

Supplemental Table 2 Primers and cyclic conditions used in PCR targeting three classes of carbapenemases genes among CRKP and CRAB clinical isolates

**carbapenemase genes**	**Primer**	**Target Gene**	**Primer sequence**	**Amplicon size (bp)**	**Cycling condition**	**Reference**
Class A	KPC	bla_{KPC}	F:GTATCGCCGTCTAGTTCTGC<br>R:GGTCGTGTTTCCCTTTAGCC	637	Initial denaturation 94°C for 5 min, then 30 cycles of 94°C for 40 s, 47°C for 1 min, 72°C for 2 min and a final extension cycle of 72°C for 5 min.	(Hong et al., 2012)
Class B	IMP	bla_{IMP}	F:GGAATAGAGTGGCTTAAYTCTC<br>R:CCAAACYACTASGTTATCT	188	Initial denaturation at 94°C for 5 min, then 30 cycles of 94°C for 40 s, 54°C for 1 min, 72°C for 2 min and a final extension cycle of 72°C for 5 min	(Ellington et al., 2006)
	VIM	bla_{VIM}	F:GATGGTGTTTGGTCGCATA<br>R:CGAATGCGCAGCACCAG	390	Initial denaturation at 94°C for 5 min, then 30 cycles of 94°C for 40 sec, 51°C for 1 min	(Ellington et al., 2006)
	NDM	bla_{NDM}	F:CACCTCATGTTTGAATTCGCC<br>R:CTCTGTCACATCGAAATCGC	984	Initial denaturation at 94°C for 5 min, then 30 cycles of 94°C for 40 s, 54°C for 1 min	(Poirel et al., 2010)
	GIM	bla_{GIM}	F:TCGACACACCTTGGTCTGAA<br>R:AACTTCCAACTTTGCCATGC	477	Initial denaturation at 94°C for 5 min, then 30 cycles of 94°C for 40 s, 52°C for 1 min	(Ellington et al., 2006)
	SPM	bla_{SPM}	F:AAAATCTGGGTACGCAAACG<br>R:ACATTATCCGCTGGAACAGG	271	Initial denaturation at 94°C for 5 min, then 30 cycles of 94°C for 40 s, 52°C for 1 min	(Ellington et al., 2006)
	SIM	bla_{SIM}	F:TACAAGGGATTCGGCATCG<br>R:TAATGGCCTGTTCCCATGTG	570	Initial denaturation at 94°C for 5 min, then 30 cycles of 94°C for 40 s, 52°C for 1 min	(Ellington et al., 2006)
Class D	OXA-23	bla_{OXA-23}	F:GATGTGTCATAGTATTCGTCGT<br>R:TCACAACAACTAAAAGCACTGT	1037	Initial denaturation 94°C for 5 min, then 30 cycles of 94°C for 50 s, 55°C for 30 s, 72°C for 45 s and a final extension cycle of 72°C for 2 min.	(Jeon et al., 2005)
	OXA-24	bla_{OXA-24}	F:ATGAAAAAATTTATACTTCCTATATTCAGC<br>R:TTAAATGATTCCAAGATTTTCTAGC	804	Initial denaturation 94°C for 5 min, then 30 cycles of 94°C for 50 s, 55°C for 30 s, 72°C for 45 s and a final extension cycle of 72°C for 2 min.	(Jeon et al., 2005)
	OXA-51	bla_{OXA-51}	F:TAATGCTTTGATCGGCCTTG<br>R:TGGATTGCACTTCATCTTGG	353	Initial denaturation 94°C for 5 min, then 30 cycles of 94°C for 50 s, 55°C for 30 s, 72°C for 45 s and a final extension cycle of 72°C for 2 min.	(Woodford et al., 2006)
	OXA-58	bla_{OXA-58}	F:AAGTATTGGGGCTTGTGCTG<br>R:CCCCTCTGCGCTCTACATAC	580	Initial denaturation 94°C for 5 min, then 30 cycles of 94°C for 50 s, 55°C for 30 s, 72°C for 45 s and a final extension cycle of 72°C for 2 min.	(Woodford et al., 2006)

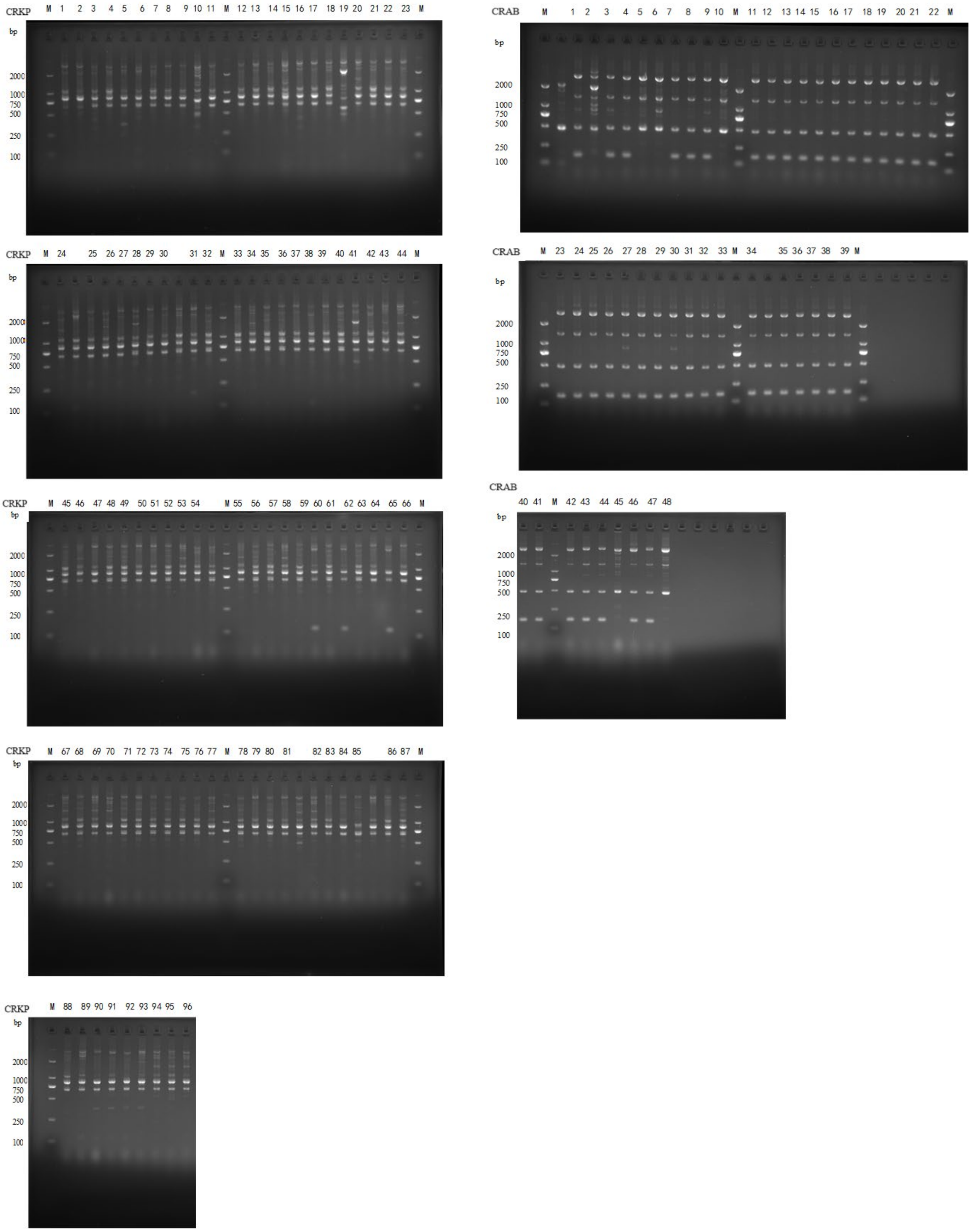

Supplemental Figure 1 Sample DNA gel image generated by REP-PCR for CRAB (1-96) and CRKP (1-48) clinical isolates.

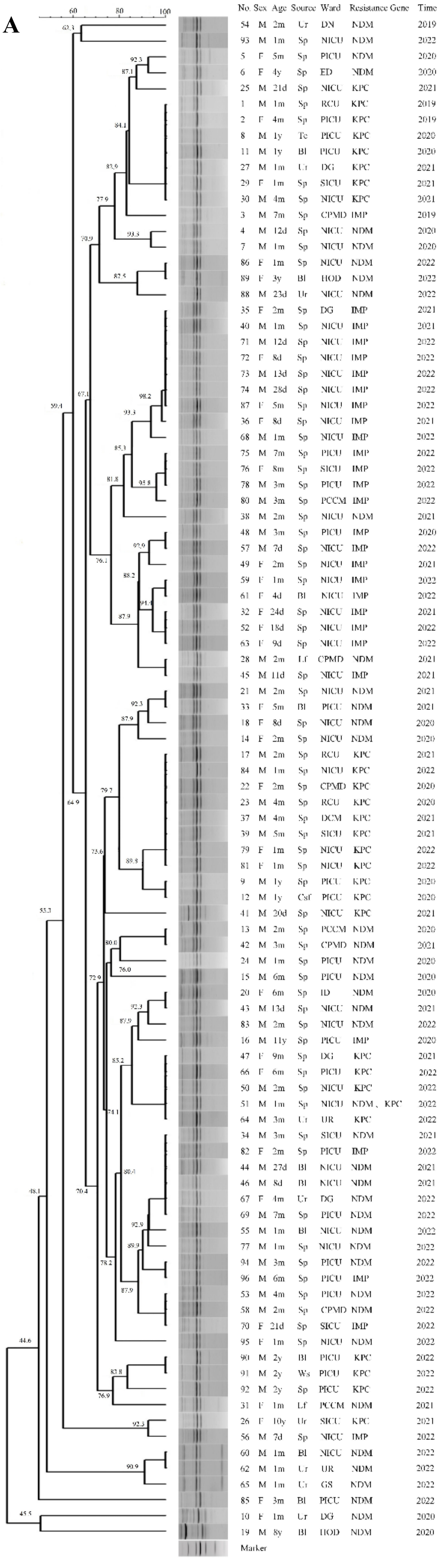

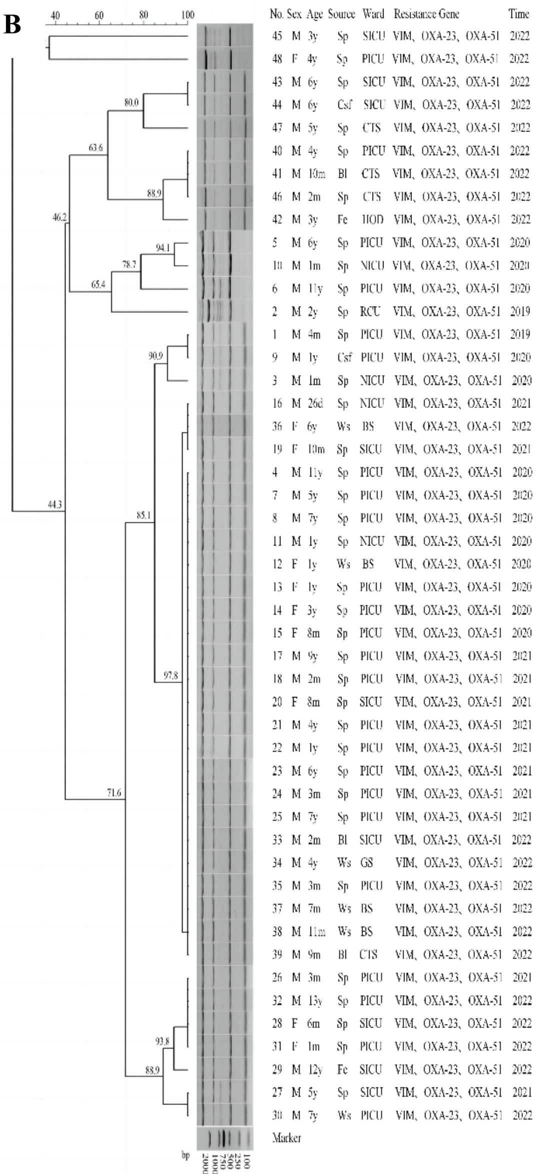

Supplemental Figure 2 Phylogenetic tree of sample gel using neighbor joining method is also shown. CRKP (A), CRAB (B).

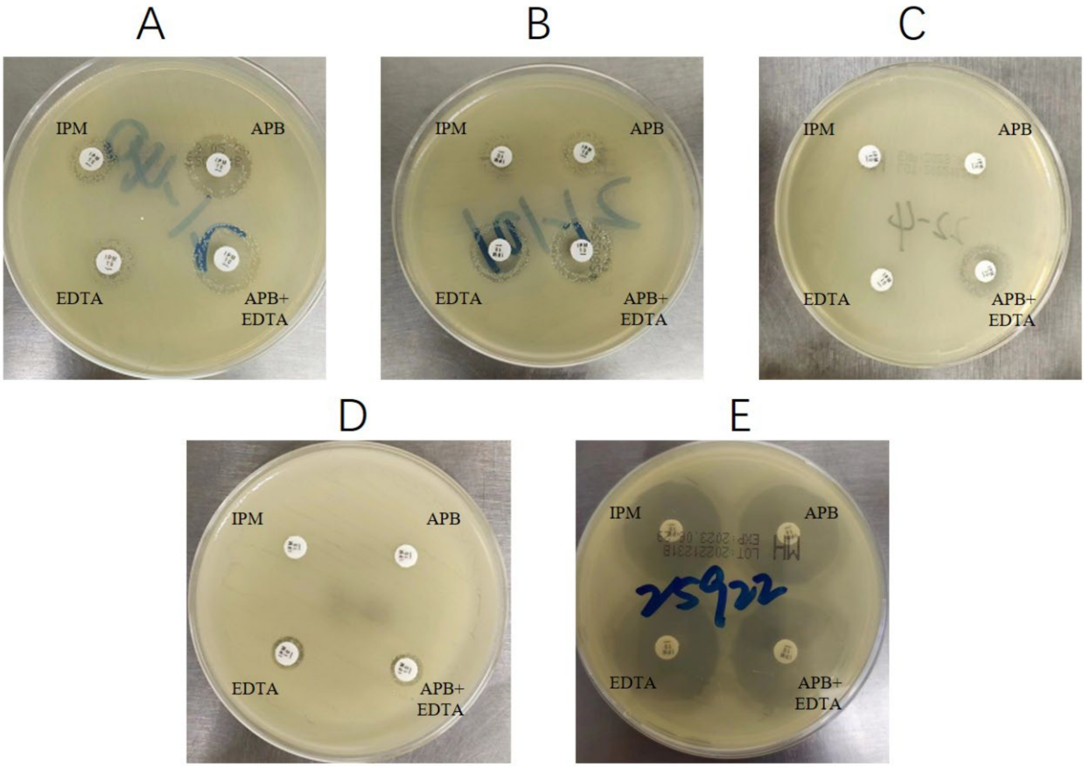

Supplemental Figure 3 Classification of carbapenemase. produce class A carbapenemase (A), produce class B carbapenemase (B), produce both class A and B carbapenemases (C), produce class D carbapenemase or extended-spectrum β-lactamases (ESBL) combined with downregulation of membrane pore proteins (D), *Escherichia coli* ATCC25922 were used as quality controls and negative for carbapenemase production (E).

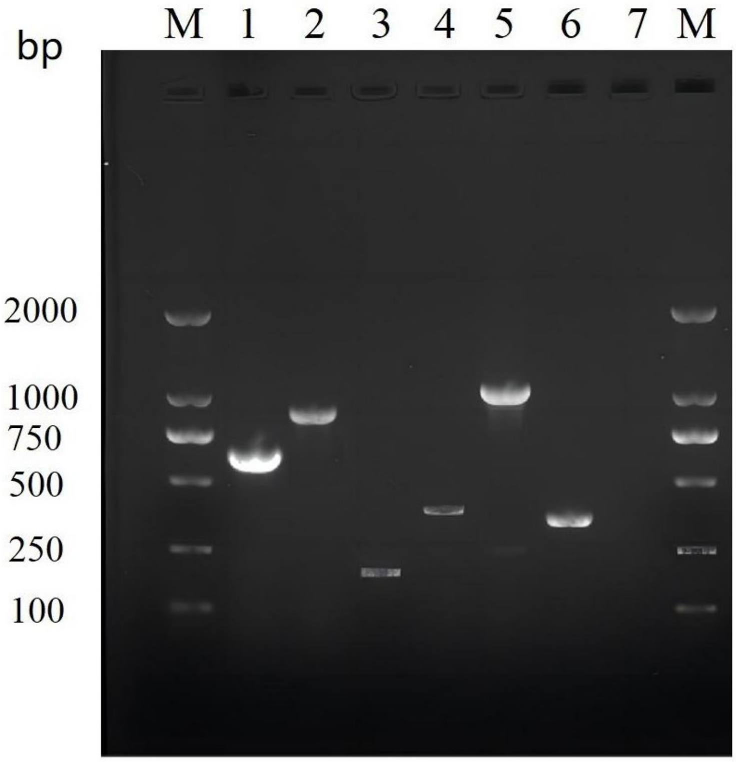

Supplemental Figure 4 Detection of carbapenemase genes bla_{KPC} (Lane 1, 637bp), bla_{NDM} (Lane2, 984 bp), bla_{IMP} (Lane 3, 188 bp), bla_{VIM} (Lane 4, 390 bp), $bla_{OXA\text{-}23}$ (Lane 5, 1037 bp), $bla_{OXA\text{-}51}$ (Lane 6, 353 bp), and negative control (Lane 7) in CRKP and CRAB isolates, using singleplex PCR and running on agarose gel.

Reference

Ellington, M.J., Kistler, J., Livermore, D.M. and Woodford, N. (2006). "Multiplex PCR for rapid detection of genes encoding acquired metallo-β-lactamases." Journal of Antimicrobial Chemotherapy 59(2): 321-322.DOI: 10.1093/jac/dkl481.

Hong, S.S., Kim, K., Huh, J.Y., Jung, B., Kang, M.S. and Hong, S.G. (2012). "Multiplex PCR for rapid detection of genes encoding class A carbapenemases." Ann Lab Med 32(5): 359-361.DOI: 10.3343/alm.2012.32.5.359.

Jeon, B.-C., Jeong, S.H., Bae, I.K., Kwon, S.B., Lee, K., Young, D., et al. (2005). "Investigation of a nosocomial outbreak of imipenem-resistant Acinetobacter baumannii producing the OXA-23 beta-lactamase in korea." Journal of clinical microbiology 43(5): 2241-2245.DOI: 10.1128/JCM.43.5.2241-2245.2005.

Poirel, L., Hombrouck-Alet, C., Freneaux, C., Bernabeu, S. and Nordmann, P. (2010). "Global spread of New Delhi metallo-β-lactamase 1." The Lancet Infectious Diseases 10(12): 832.DOI: https://doi.org/10.1016/S1473-3099(10)70279-6.

Vila, J., Marcos, M.A. and Jimenez de Anta, M.T. (1996). "A comparative study of different PCR-based DNA fingerprinting techniques for typing of the Acinetobacter calcoaceticus-A. baumannii complex." J Med Microbiol 44(6): 482-489.DOI: 10.1099/00222615-44-6-482.

Woodford, N., Ellington, M.J., Coelho, J.M., Turton, J.F., Ward, M.E., Brown, S., et al. (2006). "Multiplex PCR for genes encoding prevalent OXA carbapenemases in Acinetobacter spp." International Journal of Antimicrobial Agents 27(4): 351-353.DOI: https://doi.org/10.1016/j.ijantimicag.2006.01.004.
